# *Tiliacora triandra* Leaf Powder Ethanolic Extract in Combination with Cisplatin or Gemcitabine Synergistically Inhibits the Growth of Cholangiocarcinoma Cells In Vitro and in Nude Mouse Xenograft Models

**DOI:** 10.3390/medicina59071269

**Published:** 2023-07-07

**Authors:** Arunta Samankul, Gulsiri Senawong, Suppawit Utaiwat, Jeerati Prompipak, Khanutsanan Woranam, Chanokbhorn Phaosiri, Banchob Sripa, Thanaset Senawong

**Affiliations:** 1 Department of Biochemistry, Faculty of Science, Khon Kaen University, Khon Kaen 40002, Thailand; s_arunta@kkumail.com (A.S.); gulsiri@kku.ac.th (G.S.); u.suppawit@kkumail.com (S.U.); jeerati.ppk@kkumail.com (J.P.); khanutsanan_w@kkumail.com (K.W.); 2 Department of Chemistry, Faculty of Science, Khon Kaen University, Khon Kaen 40002, Thailand; chapha@kku.ac.th; 3 WHO Collaborating Centre for Research and Control of Opisthorchiasis (Southeast Asian Liver Fluke Disease), Tropical Disease Research Center, Faculty of Medicine, Khon Kaen University, Khon Kaen 40002, Thailand; banchob@kku.ac.th

**Keywords:** cholangiocarcinoma, cisplatin, gemcitabine, *Tiliacora triandra*, drug combination, anticancer activity

## Abstract

*Background and Objectives*: The treatments of cholangiocarcinoma (CCA) with Cisplatin (Cis) and Gemcitabine (Gem) often cause side effects and drug resistance. This study aimed to investigate the combined effects of *Tiliacora triandra* leaf powder ethanolic extract (TLPE) and Cis or Gem on CCA cells in vitro and in nude mouse xenografts. *Materials and Methods*: Antiproliferative activity was evaluated using MTT assay. Drug interaction was studied by Chou-Talalay method. Apoptosis induction and cell cycle arrest were analyzed by flow cytometry. Cell cycle and apoptosis regulating proteins were evaluated by western blot analysis. *Results*:Treatments with Cis or Gem in combination with TLPE significantly inhibited the growth of KKU-M213B and KKU-100 cells compared with single drug treatments. Synergistic drug interactions were observed with the dose reduction of Cis and Gem treatments. The safety of TLPE was demonstrated in vitro by the hemolytic assay. Synergistic combination treatments down-regulated Bcl2 and reduced the ratio of Bcl2/Bax in both CCA cells. TLPE enhanced tumor suppression of both Cis and Gem in nude mouse xenograft models. Combination treatments with Cis and TLPE reduced Cis toxicity, as demonstrated by the enhanced body weight change of the treated mice compared with the treatment with Cis alone. Furthermore, TLPE reduced hepatotoxicity caused by Gem treatment and reduced kidney and spleen toxicities caused by Cis treatment. *Conclusion*: These findings suggest that TLPE enhances the anticancer activity of Cis and Gem and reduces their toxicity both in vitro and in nude mouse xenograft models.

## 1. Introduction

Cholangiocarcinoma (CCA) arises from the cells lining the walls of the bile ducts both inside and outside the liver. CCA is divided into three types depending on its anatomical site of origin, including intrahepatic CCA, perihilar CCA, and distal CCA [1,2]. In Thailand, cholangiocarcinoma is the most established liver cancer in the northeast of the country, with 85 cases of cholangiocarcinoma per 100,000 people [3,4], which is the highest incidence in the world. Infection with *Opisthorchis viverrine* is the major risk factor for CCA in Thailand, which eventually leads to cholangiocarcinoma [3,4]. It is still difficult to diagnose this disease in its early stages since more than half of the cancer cases are diagnosed at a progressive stage of the disease with a very low survival rate. Surgical intervention may not be effective because CCA recurrence is found after surgery [5,6,7,8,9,10,11,12]. Therefore, chemotherapy is still an important treatment for slowing down the disease progression. Chemotherapy is currently popular, with several drugs being used together (combination chemotherapy) to increase the efficiency of treatment and reduce toxicity and resistance, resulting in better treatments.

The CCA chemotherapeutic drugs include Cisplatin (Cis), 5-Fluorouracil (5-FU), and Gemcitabine (Gem) [13]. Cis is a platinum coordination complex (Pt (NH_3_)_2_Cl_2_) with alkylating activity. Cis interacts with the water in the cells, giving the active form known as the “Aqua form” [14]. The aqua form can interact with DNA, where the platinum atom forms covalent bonds with the N-7 of guanine and adenosine. This process causes abnormalities in the repair and function of DNA, resulting in the death of cancer cells [15]. Gem (2’,2’-difluoro-2’-deoxycytidine) is a synthetic fluoropyrimidine analog. After Gem is absorbed into cells, a phosphate group is added by deoxycytidine kinase, a rate-limiting enzyme that activates gemcitabine. Deoxyribonucleotide inhibition of DNA polymerase [16] leads to cytotoxicity [17]. Nowadays, natural products have been shown to exert a powerful anticancer effect against CCA cells. For example, peanut testa extracts showed promising results in combination treatment with Cis against CCA cells [18]. Moreover, Thai noni juice ethanolic extract in combination with 5-FU effectively inhibited the CCA cell growth both in vitro and in nude mouse xenograft models [19].

*T. triandra*, Ya-Nang (in Thai), is a species of native flowering plants in the family of Menispermaceae, found in deciduous and dry evergreen forests of northeastern Thailand and Laos. In Thailand, Ya-nang juice is a product made from *T. triandra* leaves that have been extracted with water by hand rubbing and used as a necessary component for bamboo shoot curry and bamboo shoot salad. The bamboo shoot dishes are available in many Thai restaurants worldwide. *T. triandra* is used not only as food but also as traditional medicine. For example, it has been reported as an antipyretic, detoxication agent, anti-inflammatory agent [20], antimalarial agent [21], antimycobacterial agent [22], antioxidant [23], antidiabetic agent [24], and anticancer agent [25,26]. Several compounds identified in *T. triandra* include alkaloids [25], phenolic compounds [23,27], flavonoids, polysaccharides [27], and fatty acids [26]. A previous study demonstrated that *T. triandra* leaf water extract showed no toxicity in a single administration and no adverse effects after sub-chronic administration in rats [28]. Recently, cholangiocarcinoma cell lines were induced to undergo apoptosis after the treatment with ethanolic extract of *T. triandra* leaf powder possessing histone deacetylase (HDAC) inhibitory activity [29]. However, the combination treatments of current chemotherapeutic drugs and *T. triandra* leaf powder ethanolic extract (TLPE) have not been studied yet. In this study, the anticancer activity of TLPE in combination with current chemotherapeutic drugs (Cis and Gem) was investigated against CCA cells both in vitro and in nude mouse xenograft models. The mechanisms underlying the anticancer activity were determined as well.

## 2. Materials and Methods

### 2.1. Materials

*T. triandra* leaf powder (Lot no. 12/01/2019) was ordered from the Rak Samunpai group, Bangkok, Thailand. The product is approved by the Food and Drug Administration of Thailand (Thai FDA) with FDA number and product identification numbers: 10-1-12958-1-0005 and 4137141, respectively. Cisplatin, Gemcitabine, propidium iodine (PI), and Triton X-100 were acquired from Sigma-Aldrich Corporation (St. Louis, MO, USA). Gemcitabine for the in vivo study was purchased from Tokyo Chemical Industry Co., Ltd. (Tokyo, Japan). The MTT was purchased from Invitrogen, Molecular Probes products (Eugene, OR, USA), whereas the Annexin V-FITC was obtained from Biolegend (San Diego, CA, USA).

### 2.2. Cell Lines and Culture Conditions

KKU-M213B and KKU-100 (well-differentiated and poorly differentiated/drug-resistant, respectively) cell lines were previously established [30,31]. The cholangiocarcinoma cells were cultured as previously described [31]. H69 (a non-cancer cell line; human bile duct epithelial) cells were obtained from Dr. D. Jefferson (Tufts University, Boston, MA, USA) [32] and cultured as previously described [18]. KKU-M213B and KKU-100 cells were cultured in RPMI-1640 supplemented with 10% fetal bovine serum and antibiotics (100 U/mL penicillin and 100 μg/mL streptomycin (Gibco, New York, NY, USA), whereas H69 cells were maintained in Dulbecco’s minimum essential medium (DMEM; Gibco, New York, NY, USA) supplemented with 10% FBS, 100 μg/mL streptomycin, 100 U/mL penicillin, 25 μg/mL adenine, 10 ng/mL EGF, 1 μg/mL epinephrine, 8.30 μg/mL holo-transferrin, 0.62 μg/mL hydrocortisone, 5 μg/mL insulin, and 13.60 ng/mL T3T Triiodo-L-thyronine. All cell lines were maintained in our laboratory in >100 passages over the past 10 years.

### 2.3. Preparation of T. triandra Leaf Powder Ethanolic Extract (TLPE)

TLPE was prepared to evaluate the total phenolic content and antiproliferative activity. The effects of TLPE on cell cycle progression, induction of apoptosis, levels of regulatory protein expression, and antitumor activity in nude mouse xenograft models were also tested. *T. triandra* leaf powder was added with absolute ethanol (1:10) and extracted as previously described [29]. Briefly, 20 g of *T. triandra* leaf powder was added in 200 mL of absolute ethanol, stirred for 48 h, and then centrifuged at 6, 150× *g* (Hitachi centrifuge with the R20A2 rotor) for 15 min. The supernatant was collected and filtered through a Whatman No. 4 filter paper, evaporated to 2 mL using a rotary evaporator, and finally to dryness under a gentle stream of nitrogen. TLPE was kept at −20 °C until it was used. The extraction yield was 5.41 ± 0.61%.

### 2.4. Preparation of Phenolic Extract for the Analysis of Phenolic Acids

A phenolic extract of *T. triandra* leaf powder was produced to obtain free polar and nonpolar phenolic compounds. *T. triandra* leaf powder (20 g) was macerated with absolute ethanol (200 mL) for 48 h. The suspension was centrifuged for 15 min at 10,000 rpm. The supernatant was filtered through a Whatman No. 4 filter paper. The filtrate was added with 40 mL of double distilled water and evaporated to 40 mL using a rotary evaporator. Then, the filtrate was extracted further, as previously described [33,34]. Briefly, the filtrate was added with 40 mL of 2 N NaOH, stirred continuously for 12 h at room temperature, and then centrifuged at 1700× *g* for 10 min. The supernatant was filtered through a Whatman grade No.1 filter paper and repeatedly extracted three times with 40 mL of diethyl ether. The aqueous phase was collected and adjusted to pH 1.5 by 10 N HCl. The aqueous phase was filtered through a Whatman grade No.1 filter paper and extracted with 40 mL of diethyl ether three times using a separating funnel. The portion of diethyl ether was collected. The diethyl ether phase was added with sodium sulfate (Na_2_SO_4_) anhydrous and filtered through a Whatman grade No.1 filter paper. The filtrate was evaporated to 2 mL using a rotary evaporator and finally evaporated to dryness under a gentle stream of nitrogen. Finally, the phenolic extract was kept at −20 °C until it was used. The extraction yield was 0.13 ± 0.04%. The phenolic extract was analyzed for phenolic acid compositions using the HPLC technique described below in Section 2.5.

### 2.5. Phenolic Acid Compositions of TLPE

Specific phenolic acids within the extract were identified using reversed-phase HPLC. The phenolic acids were identified based on matching the retention times and the spectrum to that of phenolic acid standards as previously described [34]. The peak areas were used to analyze the concentration of each phenolic acid. The standard phenolic acids (caffeic, *p*-coumaric, ferulic, gallic, *p*-hydroxybenzoic, protocatechuic, sinapinic, syringic, and vanillic acids) and the internal standard (*m*-hydroxybenzaldehyde) were used for identification of phenolic compounds in TLPE.

### 2.6. Total Phenolic Contents of TLPE

Total phenolic content was determined as described in the previously established protocols [34] that rely on the reduction of phosphomolybdic and phosphotungstic acids (Folin-Ciocalteu reagent) by the hydroxyl groups of phenolic compounds. The microplate reader (Bio-Rad Laboratories, Hercules, CA, USA) was then used to measure the absorbance at 750 nm. Total phenolic content was calculated using a gallic acid standard curve and reported as the gallic acid equivalent per gram of dry TLPE.

### 2.7. Antiproliferative Activity Assay

Antiproliferative activity was determined using MTT (3-(4,5-dimethylthiazol-2yl) 2,5-diphenyl tetrazolium bromide) assay as described previously [35]. The cells were treated with various concentrations of single agent (Cis, Gem, and TLPE) and a solvent control (0.5% ethanol + 0.5% dimethyl sulfoxide (DMSO)) for 24, 48, and 72 h. The combination treatments with TLPE (1.90–1000 µg/mL final concentrations) and Cis or Gem (sub-toxic concentration; IC_20_) were performed at 24, 48, and 72 h exposures. The following equation was used to calculate the cell viability:

$$\%\;Cell\;viability=\frac{A550\;Sample-O.D.655\;Control}{A550\;Control-O.D.655\;Control}\times 100$$

where *A* and *O.D*. are the absorbance and optical density, respectively.

### 2.8. Hemolytic Activity

To evaluate the hemolytic toxicity of the TLPE, standard procedures for conducting a hemolytic assay were used [36]. Khon Kaen University’s Ethics Committee for Human Research reviewed the experimental protocols based on the Declaration of Helsinki and the ICH Good Clinical Practice Guidelines (in order of 3.4.02: 11/2022 No. HE652002). Briefly, red blood cells (RBCs) were isolated from the blood of healthy volunteers (25–30 years old) who had not smoked, drank alcohol, or taken any drugs in the previous month. RBCs were washed with 1X PBS 3 times and centrifuged at 1500 rpm. RBCs (100 µL of 4% RBCs in a 96-well plate) were incubated with Cis (25–200 µg/mL), Gem (250–2000 µg/mL), and TLPE (62.5–1000 µg/mL) at 37 °C and 5% CO_2_ for 1 h. Triton X-100 (1%), as well as phosphate-buffered saline (PBS), were utilized as positive and negative controls, respectively. RBCs were subjected to centrifugal force at 1500 rpm at 4 °C for 10 min, and the supernatants were then collected. The absorbance was measured at 540 nm. The following formula was used to calculate the hemolytic activity:

$$\%\;Hemolytic\;activity=\frac{A540\;Sample-A540\;Negative\;control}{A540\;Positive\;Control}\times 100$$

where *A* is the absorbance.

### 2.9. Determination of Drug Interaction

The Chou–Talalay method [37] was used to estimate the combination index (*CI*) and to assess the type of drug interaction between Cis or Gem and TLPE. The following is the equation of *CI* values for 50% growth inhibition:

$$CI=\left(\frac{D1}{Dx1}\right)+\left(\frac{D2}{Dx2}\right)+\alpha \frac{D1D2}{Dx1Dx2}$$

where *D*1 is a dose of drug 1 (Cis or Gem) combined with drug 2 (TLPE) to produce 50% cell viability; *Dx*1 is a dose of single drug 1 to produce 50% cell viability; *D*2 is a dose of drug 2 combined with drug 1 to produce 50% cell viability; *Dx*2 is a dose of single drug 2 to produce 50% cell viability; α = 1 for mutually non-exclusive modes of drug action. *CI* < 0.90 indicates a synergistic effect; *CI* = 0.90–1.10 indicates an additive effect, and *CI* > 1.10 indicates antagonism. The dose reduction index (*DRI*) indicates the extent of dose reduction (fold) of the combined dose tested compared to the dose of a single agent. The *DRI* was calculated using the following equation:

$$DRI=\left(\frac{Dx}{D}\right)$$

where D is a dose of a drug combined with the other drug to produce 50% cell viability; Dx is a dose of a single drug to produce 50% cell viability.

### 2.10. Apoptosis Analysis by Flow Cytometry

Annexin-V FITC (green fluorescence) and propidium iodide (red fluorescence) staining were used to detect apoptotic cells, as previously described [35]. KKU-100 and KKU-M213B cell lines were seeded at a density of 1 × 10^6^ cells/5.5 cm dish plate and incubated for 24 h. Synergistic concentrations of TLPE, Cis, and Gem were used to treat KKU-M213B cells for 24 and 48 h and used to treat KKU-100 cells for 48 and 72 h, respectively. The solvent control was a mixture of 0.5% ethanol and 0.5% DMSO. Trypsinization was used to harvest the cells that had been treated, and then they were centrifuged at 4000 rpm for 3 min and washed twice with ice-cold 1X PBS. Annexin-binding buffer (pH 7.4) was used to resuspend the cells before staining with Annexin-V FITC and PI. The BD FACSCanto II flow cytometer (Becton Dickinson, San Jose, CA, USA) was used to examine the stained cells after incubating the cells in the dark for 15 min at room temperature.

### 2.11. Cell Cycle Analysis by Flow Cytometry

Propidium iodide (PI) staining was used to determine cell cycle arrest, as described previously [35]. Synergistic concentrations of TLPE, Cis, and Gem were used to treat KKU-M213B cells for 24 and 48 h and used to treat KKU-100 cells for 48 and 72 h, respectively. The BD FACSCanto II flow cytometer (Becton Dickinson, San Jose, CA, USA) was used to analyze the stained cells.

### 2.12. Western Blot Analysis

To analyze the expression of apoptosis-related proteins and ERK signaling proteins, KKU-M213B cells were exposed to TLPE, Cis, or Gem alone or in a synergistic combination for 24 h and 48 h, respectively. KKU-100 cells were treated with TLPE, Cis, and Gem alone or in combination at a synergistic concentration for 48 h and 72 h, respectively. The solvent control was a mixture of 0.5% ethanol and 0.5% DMSO. RIPA lysis buffer (Amresco, Solon, OH, USA) with a protease inhibitor cocktail was used to extract the protein. The protein content was measured using the Bio-Rad protein assay (Bio-Rad, Hercules, CA, USA). The samples with equal amounts of protein were added with 2X loading buffer containing β-mercaptoethanol, subsequently boiled in boiling water, resolved by SDS-PAGE (12.5%), and transferred to the polyvinylidene fluoride (PVDF) membrane. The blots were blocked with 3% skim milk in PBS-T for 30 min and then incubated with primary antibody against Bcl-2 (#2870, diluted 1:1000), Bax (#2772, diluted 1:1000), pERK1/2 (#4377, diluted 1:1000), Ac-H3 (#9649, diluted 1:2000), or ERK1/2 (#9107, diluted 1:2000) (Cell Signaling, Danvers, MA, USA) at 4 °C overnight. Thereafter, the blots were washed with PBS-T before incubating for 2 h with anti-mouse (#7076, diluted 1:2000) or anti-rabbit (#7074, diluted 1:2000) conjugated horseradish peroxidase secondary antibody at room temperature. The blots were washed with PBS-T and PBS, respectively. The blots were then developed with a chemiluminescence reagent (Bio-Rad, CA, USA) before exposing the immunoreactive bands to the X-ray film. The loading control was total ERK1/2 to allow for the relative intensity to be measured.

### 2.13. Antitumor Activity in Nude Mouse Xenograft Model

Female nude mice (BALB/CAJcl-Nu/Nu) were obtained from Nomura Siam International (Bangkok, Thailand) at the age range of 6–7 weeks. The animal experiments were approved by Khon Kaen University’s Institutional Animal Care and Use Committee (IACUC-KKU-106/63; date of registration 7 January 2021) and performed in accordance with guidelines established by the National Research Council of Thailand’s Ethical Principles and Guidelines for the Use of Animal in Scientific Purposes. The study was conducted in accordance with the ARRIVE guidelines. Animals were housed and maintained in an environment devoid of specific pathogens with 12 h light: 12 h dark cycles at 23 ± 2 °C at the Northeast Laboratory Animal Center, Khon Kaen University, Khon Kaen, Thailand. After acclimatization for 7 days, 30 mice received KKU-100 cells via subcutaneous injection into the right axilla. The dose was 5 × 10^6^ cells in 100 mL of serum-free media mixed with Matrigel (Corning, Tewksbury, MA, USA). The following formula was used to determine tumor volume from measurements taken using a digital vernier caliper:

$$Tomor\;volume=\frac{length\times {width}^{2}}{2}$$

Mice with tumor volumes of 100 mm^3^ were divided at random into 6 groups (n = 5 per group; sample size calculation performed by two independent means (independent *t*-test)): (1) vehicle control (DMSO + 1X PBS (1:9)), (2) Cis (received 3 mg/kg Cis), (3) Gem (received 100 mg/kg Gem), (4) TLPE (received 100 mg/kg TLPE), (5) TLPE + Cis (received 100 mg/kg TLPE and 3 mg/kg Cis), and (6) TLPE + Gem (received 100 mg/kg TLPE and 100 mg/kg Gem). Mice were treated by i.p. injection every 3 days for 18 days. The body weight and tumor volume were recorded every 3 days for 21 days before the mice were sacrificed. The xenograft tumor, kidney, liver, and spleen were collected and weighed 3 days after the final treatment was administered. The following formula was used to determine the relative tumor volume (*RTV*):

$$RTV=\frac{tumor\;volume\;on\;the\;measured\;day}{tumor\;volume\;on\;day\;0}$$

The tumor growth inhibition ratio (*TGI*, %) was determined as follows:

$$\%\;TGI=\left(1-\frac{RTV\;in\;experimental\;group}{RTV\;in\;control\;group}\right)\times 100$$

The final step was an analysis of the toxicity based on the body weight change (*BWC*):

$$\%\;BWC=\frac{body\;weight\;on\;measured\;day-body\;weight\;on\;day\;0}{body\;weight\;on\;day\;0}\times 100$$

### 2.14. Histopathology

To determine and characterize the histological changes induced in the tissues, the liver, kidneys, and spleen were fixed in 10% formalin for 48–96 h. Then, the organs were excised, dehydrated, and cleaned in tissue processing for 12 h. The samples were embedded in paraffin, then cut with a microtome to a thickness of 4 µm. The tissue sections were mounted on a glass slide and deparaffinized in xylene, rehydrated in 99, 95, and 70% ethanol, respectively, and finally washed in distilled water. Hematoxylin and eosin were used to stain the rehydrated tissue samples. Thereafter, the samples were examined under a light microscope for any anomalies that emerged.

### 2.15. Statistical Analysis

The data were presented using a mean ± standard deviation (SD) from at least two independent experiments. The data were analyzed using the Statistical Package for the Social Science version 25.0 for Windows (SPSS Corporation, Chicago, IL, USA). The significant differences were analyzed using one-way ANOVA with Duncan’s post hoc test, and the *p* < 0.05 was referred to as statistically significant.

## 3. Results

### 3.1. Phenolic Contents and Phenolic Acid Compositions of TLPE

**Figure 1:**
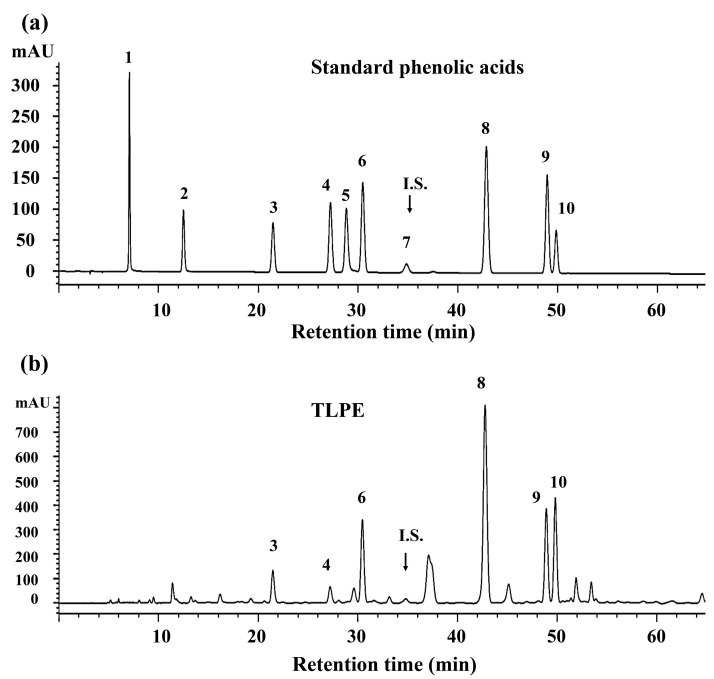
HPLC chromatogram of standard phenolic acids (**a**) and *T. triandra* leaf powder ethanolic extract (**b**):1, gallic acid; 2, protocatechuic acid; 3, *p*-hydroxybenzoic acid; 4, vanillic acid; 5, caffeic acid; 6, syringic acid; 7, *m-*hydroxybenzaldehyde (internal standard; I.S.); 8, *p*-coumaric acid; 9, ferulic acid and 10, sinapinic acid.

The total phenolic content of the TLPE was 42.51 ± 6.84 mg gallic acid equivalent/g of dry extract. The phenolic acid compositions were analyzed using HPLC, as shown in Figure 1 and Table 1. Six types of phenolic acids can be identified: (1) *p*-coumaric, (2) ferulic, (3) *p*-hydroxybenzoic, (4) sinapinic, (5) syringic, and (6) vanillic acids. Among the six identified phenolic acids, sinapinic acid (9.98 ± 2.25 mg/g) was found to be the predominant phenolic compound in TLPE.

### 3.2. Antiproliferative Effects of the Single Agent Treatment of Cis, Gem, and TLPE

**Figure 2:**
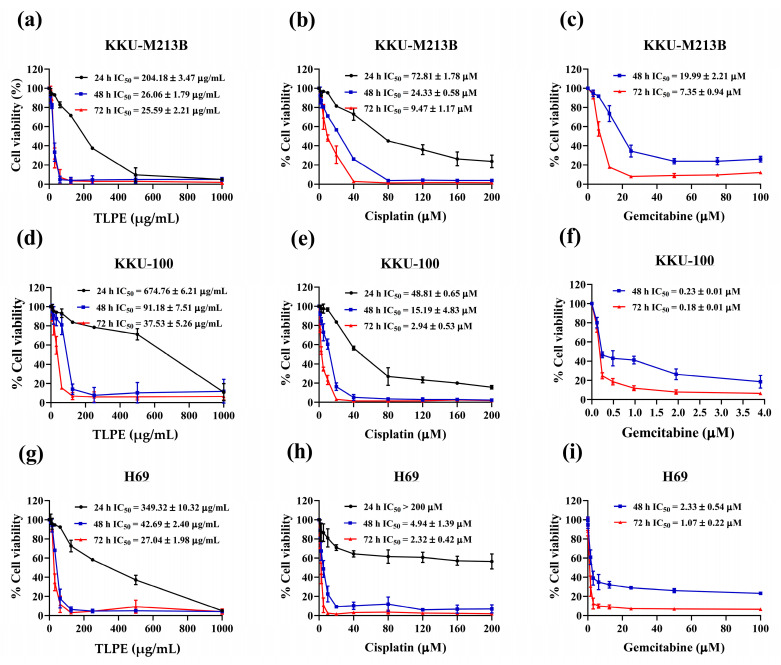
Antiproliferative effects of the single treatments. KKU-M213B (**a**–**c**), KKU-100 (**d**–**f**), and H69 (**g**–**i**) cells were treated with TLPE, Cis, and Gem for 24, 48, and 72 h at the indicated concentrations.

The dose- and time-dependent inhibitions of cell proliferation by Cis, Gem, and TLPE were observed (Figure 2). Evaluation of cellular sensitivity and assessment of the efficacy of the combination therapies were mostly based on the half-maximal inhibitory concentration (IC_50_) values of each individual drug. TLPE inhibited the proliferation of KKU-M213B cells with IC_50_ values of 204.18 ± 3.47, 26.06 ± 1.79, and 25.59 ± 2.21 µg/mL at the exposure times of 24, 48, and 72 h, respectively (Figure 2a). Cis inhibited the proliferation of KKU-213B cells with IC_50_ values of 72.81 ± 1.78, 24.33 ± 0.58, and 9.47 ± 1.17 µM for 24 h, 48 h, and 72 h exposures, respectively (Figure 2b).

In addition, Gem suppressed the proliferation of KKU-M213B cells with IC_50_ values of 19.99 ± 2.21 and 7.35 ± 0.94 µM at the exposure times of 48 and 72 h, respectively (Figure 2c). In the meantime, TLPE restrained the proliferation of KKU-100 cells with IC_50_ values of 674.76 ± 6.21, 91.18 ± 7.51, and 37.53 ± 5.26 µg/mL for 24 h, 48 h, and 72 h exposures, respectively (Figure 2d). The IC_50_ values of Cis against KKU-100 cells at the exposure times of 24, 48, and 72 h were 48.81 ± 0.65, 15.19 ± 4.83, and 2.94 ± 0.53 µM, respectively (Figure 2e). Gem inhibited the growth of KKU-100 cells with the IC_50_ values of 0.23 ± 0.01 and 0.18 ± 0.01 µM for 48 h and 72 h exposures, respectively (Figure 2f). Furthermore, the IC_50_ values of TLPE against a non-cancer cell line (H69) were 349.32 ± 10.32, 42.69 ± 2.40, and 27.04 ± 1.98 µg/mL for 24 h, 48 h and 72 h exposures, respectively (Figure 2g). Cis inhibited the proliferation of H69 cells with IC_50_ values of >200, 4.94 ± 1.39, and 2.32 ± 0.42 µM for 24 h, 48 h, and 72 h exposures, respectively (Figure 2h). Gem reduced the viability of H69 cells with IC_50_ values of 2.33 ± 0.54 and 1.07 ± 0.22 µM for 48 h and 72 h exposures, respectively (Figure 2i). The IC50 values of Gem against KKU-M213B, KKU-100, and H69 cells at the exposure time of 24 h were >1000 µM (Supplementary Figure S1).

### 3.3. Hemolytic Toxicity of Cis, Gem, and TLPE by Hemolytic Assay

**Figure 3:**
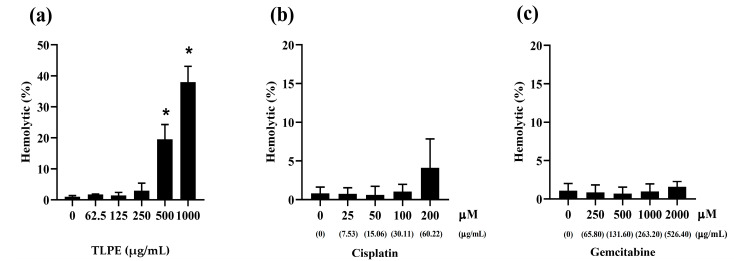
Hemolytic effects of TLPE (**a**), Cis (**b**), and Gem (**c**) on human red blood cells using Triton X-100 as a positive control and 1X PBS as a negative control when incubated at 37 °C for 1 h. “*” denotes a statistically significant difference (*p* < 0.05) compared to the solvent control treatment.

The hemolytic effects of Cis, Gem, and TLPE on human red blood cells were determined using Triton X-100 as a positive control and 1X PBS as a negative control. The treatments with the highest concentrations of Cis (200 µM), Gem (2000 µM), and TLPE (1000 µg/mL) caused hemolysis of red blood cells less than 50% (Figure 3a–c). Specifically, TLPE at 250 µg/mL caused hemolysis of red blood cells less than 5% (Figure 3a).

### 3.4. Combination Index and Dose Reduction Index of Cis or Gem and TLPE in Combination Treatments

According to the Chou–Talalay method [37], the combination index (CI) and dose reduction index (DRI) values were calculated to evaluate the nature of pharmacological interactions between TLPE and Cis or Gem. In the combination treatments with different doses of TLPE, the sub-toxic dose (the concentration at which growth inhibition is 20%; IC_20_) of Cis or Gem was fixed for each CCA cell line. In KKU-M213B cells, the combination treatments of Cis with TLPE at 24 h exposure and Gem with TLPE at 48 h exposure showed CI values less than 0.90, indicating a synergistic effect (Table 2). However, the combination treatments of TLPE with both Cis and Gem at 72 h exposure revealed an antagonistic effect in KKU-M213B cells. The combination treatments of TLPE with Cis at 48 h exposure and with Gem at 72 h exposure exhibited a synergistic effect in KKU-100 cells (Table 2). In contrast, TLPE combined with Cis at 72 h exposure and with Gem at 48 h exposure showed an additive effect (CI ~ 1.0) in KKU-100 cells. In addition, the synergistic effect of TLPE and Cis at 48 h exposure in KKU-M213B cells resulted in the greatest dose reduction (4.63-fold) for Cis, while the synergistic effect of TLPE and Gem at 48 h exposure resulted in the greatest dose reduction (8.65-fold) for TLPE. In KKU-100 cells, the synergistic effect of TLPE combined with Cis at 48 h exposure resulted in the greatest dose reduction (4.05-fold) for Cis, whereas the synergistic effect of TLPE with Gem at 72 h exposure demonstrated the greatest dose reduction (17.54-fold) for TLPE.

### 3.5. Antiproliferative Effects of the Synergistic Combination Treatments of TLPE and Cis or Gem against CCA Cell Lines

**Figure 4:**
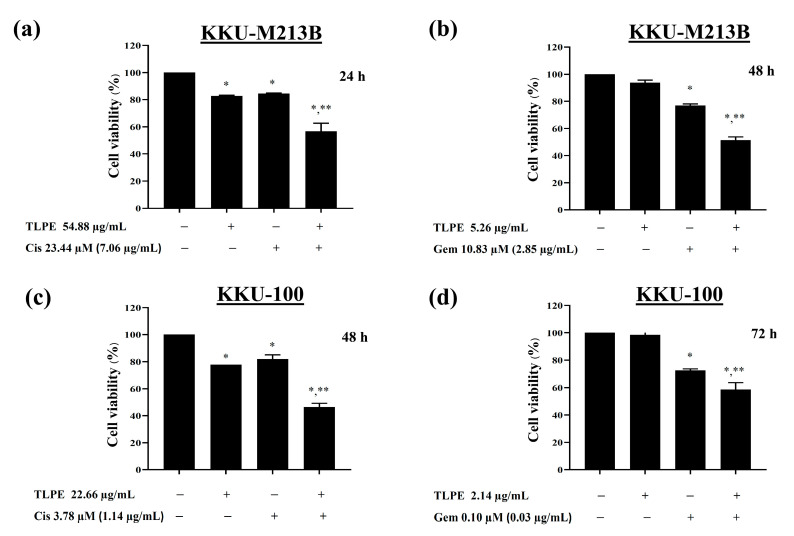
Antiproliferative effects of the combination treatment between TLPE and Cis or Gem. KKU-M213B (**a**,**b**) and KKU-100 (**c**,**d**) cells were treated with TLPE at a synergistic concentration and Cis or Gem at a sub-toxic concentration. Cell viability was calculated as a percentage in comparison to cells treated with solvent control (0.50% ethanol + 0.50% DMSO). “*” denotes a statistically significant difference (*p* < 0.05) compared to the solvent control treatment. “**” denotes a statistically significant difference (*p* < 0.05) compared to the single-agent treatments.

The combined effects of Cis or Gem at a sub-toxic dose (IC_20_) with the synergistic concentrations of TLPE against CCA cell lines were demonstrated comparatively with the single drug treatments (Figure 4). Cis alone (sub-toxic concentration) reduced viabilities of KKU-M213B and KKU-100 cells to 84.42 ± 0.44% (24 h exposure) and 81.89 ± 3.17% (48 h exposure), respectively (Figure 4a,c). TLPE alone (synergistic concentration with Cis) reduced KKU-M213B and KKU-100 viabilities to 82.08 ± 0.51% (24 h exposure) and 75.57 ± 3.09% (48 h exposure), respectively (Figure 4a,c).

Combination treatments of Cis and TLPE significantly reduced the proliferation of KKU-M213B (Figure 4a) and KKU-100 cells to 56.78 ± 5.91% and 46.38 ± 2.77% (Figure 4c), respectively. Gem alone (sub-toxic concentration) reduced KKU-M213B and KKU-100 viabilities to 76.87 ± 1.20% (48 h exposure) and 73.37 ± 2.36% (72 h exposure), respectively (Figure 4b,d). TLPE alone (synergistic concentration with Gem) reduced KKU-M213B and KKU-100 viabilities to 93.51 ± 8.9% (48 h exposure) and 98.50 ± 6.11% (72 h exposure), respectively (Figure 4b,d). Combination treatments of Gem and TLPE significantly reduced the proliferation of KKU-M213B (Figure 4b) and KKU-100 (Figure 4d) cells to 51.48 ± 2.38% and 58.55 ± 5.18%, respectively.

### 3.6. Effect of the Combination Treatment of TLPE and Cis or Gem on Cell Cycle Progression and Apoptosis Induction

**Figure 5:**
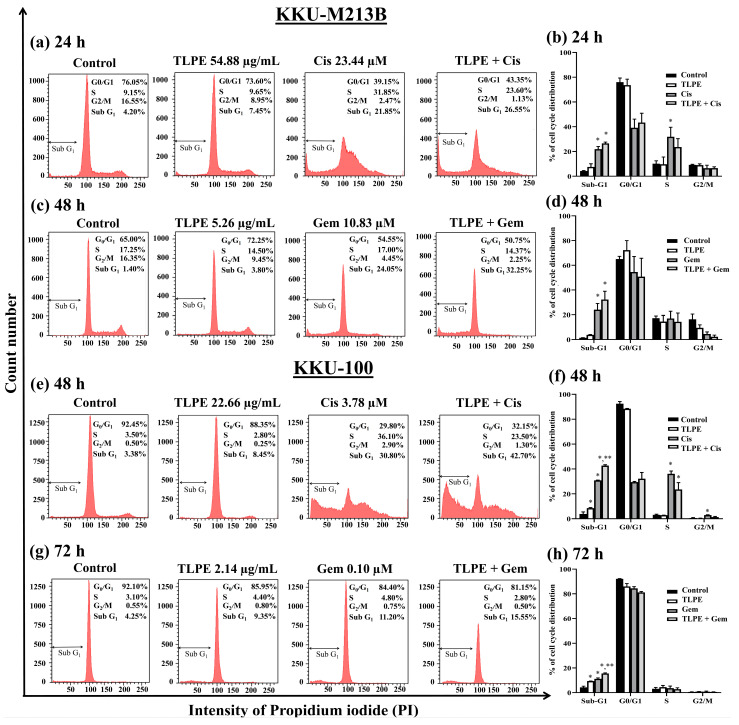
Effect of the combination treatments on cell cycle distribution. Histograms show the distribution of KKU-M213B (**a**,**c**) and KKU-100 (**e**,**g**) cells according to the DNA content after being treated with TLPE, Cis, and Gem alone and in combination. The treatment with 0.5% EtOH + 0.5% DMSO (*v/v*) was used as a solvent control. Bar graphs show the summarized data (means ± S.D.) of KKU-M213B (**b**,**d**) and KKU-100 (**f**,**h**) cell cycle profiles from two-three independent experiments performed in duplicate. “*” denotes a statistically significant difference (*p* < 0.05) compared to the solvent control treatment. “**” denotes a statistically significant difference (*p* < 0.05) compared to the single-agent treatments.

Cell cycle arrest and apoptosis induction in the combination treatments of TLPE and Cis or Gem were investigated. The dose and exposure time at the synergistic condition were used to assess the cell cycle arrest and apoptosis induction in both cells (Figure 5). In KKU-M213B cells, Cis alone (IC_20_; 23.44 µM) caused an increase of sub-G1 population (21.85 ± 2.19%). In contrast, TLPE alone at the synergistic concentration (54.88 µg/mL) did not affect any cell cycle phase in KKU-M213B cells. However, TLPE alone caused a slight increase in the sub-G1 population (7.45 ± 2.62%). Combination treatment of Cis and TLPE caused more increased sub-G1 population (26.55 ± 1.06%) than the single treatments of Cis and TLPE (Figure 5a,b). In addition, Gem alone (IC_20_; 10.83 µM) promoted apoptosis as the sub-G1 population was increased (24.05 ± 5.02), while TLPE alone (5.26 µg/mL) caused a slight increase in the sub-G1 population (3.80 ± 0.28%) in KKU-M213B cells. The combination treatment of Gem and TLPE caused more increased sub-G1 population (32.25 ± 6.72%) than the single treatments of Cis and TLPE (Figure 5c,d) in KKU-M213B cells. In KKU-100 cells, Cis alone (IC_20_; 3.78 µM) caused an increased sub-G1 population (30.80 ± 0.28%), whereas TLPE alone (22.66 µg/mL) caused a slight increase in sub-G1 population (8.45 ± 0.49%) (Figure 5e,f). The combination treatment of Cis and TLPE significantly increased the sub-G1 population (42.70 ± 0.71%) in KKU-100 cells (Figure 5e,f). Both Gem alone (IC_20_; 0.10 µM) and TLPE alone (2.14 µg/mL) induced apoptosis as shown by the increased sub-G1 populations (11.20 ± 0.71 and 9.35 ± 0.07%, respectively) (Figure 5g,h). Furthermore, combined Gem and TLPE significantly increased the sub-G1 populations (15.55 ± 0.49%) (Figure 5g,h).

**Figure 6:**
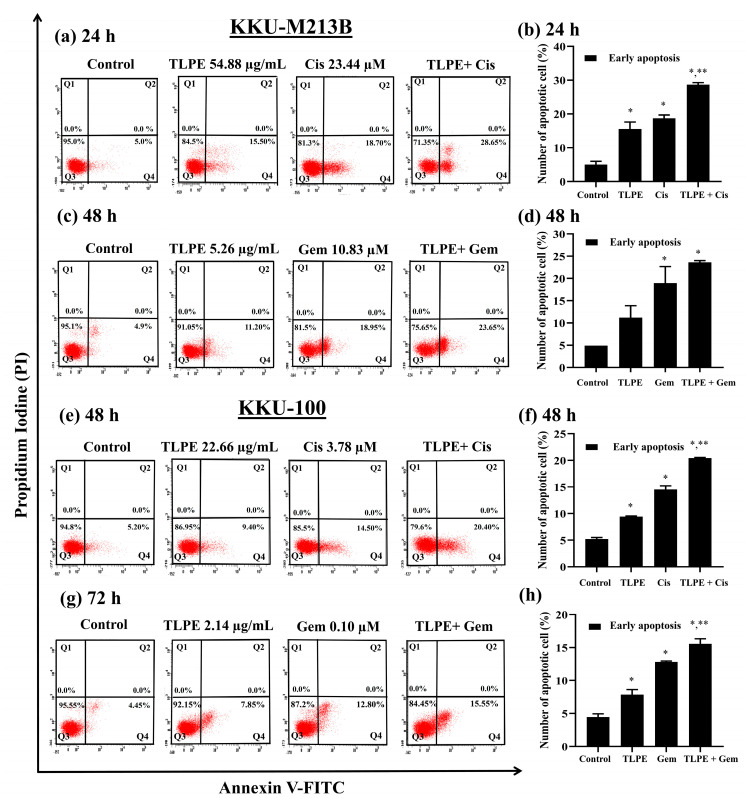
Effect of the combination treatments under the synergistic conditions on induction of apoptosis. The representative dot plots of KKU-M213B (**a**,**c**) and KKU-100 (**e**,**g**) cells are shown. The percentages of cells undergoing early (panels Q4) and late (panels Q2) apoptosis were analyzed using flow cytometry after staining the cells with Annexin V-FITC and propidium iodide (PI). The treatment with 0.5% EtOH + 0.5% DMSO (*v/v*) was used as a solvent control. Bar graphs represent the summarized data (means ± S.D.) on apoptosis induction in KKU-M213B (**b**,**d**) and KKU-100 (**f**,**h**) cells from two-three independent experiments performed in duplicate. “*” denotes a statistically significant difference (*p* < 0.05) compared to the solvent control treatment. “**” denotes a statistically significant difference (*p* < 0.05) compared to the single-agent treatments.

Apoptotic induction by the combination treatment at a synergistic condition of Cis and TLPE in CCA cell lines was demonstrated in Figure 6. Combined Cis and TLPE significantly induced apoptosis in KKU-M213B cells (28.65 ± 0.64%). The single-agent treatments of Cis and TLPE demonstrated less apoptotic induction (18.70 ± 0.99%, and 15.5 ± 2.12%, respectively) (Figure 6a,b). Combination treatment at a synergistic condition of Gem and TLPE significantly induced apoptosis in KKU-M213B cells (23.65 ± 0.35%). Meanwhile, the single-agent treatments of Gem and TLPE caused less apoptotic induction (18.95 ± 3.75% and 11.2 ± 2.69%, respectively) (Figure 6c,d). Similarly, the combination treatment at a synergistic condition of Cis and TLPE significantly induced apoptosis (20.40 ± 0.14%) in KKU-100 cells, while the single-agent treatments of Cis and TLPE caused less apoptotic induction (14.50 ± 0.71% and 9.40 ± 0.14%, respectively) (Figure 6e,f). In addition, the combination treatment at a synergistic condition of Gem and TLPE caused significant induction of apoptosis in KKU-100 cells (15.55 ± 0.78%), whereas both Cis alone and TLPE alone induced less apoptosis (12.8 ± 0.14% and 7.85 ± 0.78%, respectively) (Figure 6g,h).

### 3.7. Effect of the Combination Treatment of TLPE and Cis or Gem on Apoptosis-Related Proteins and ERK Signaling

**Figure 7:**
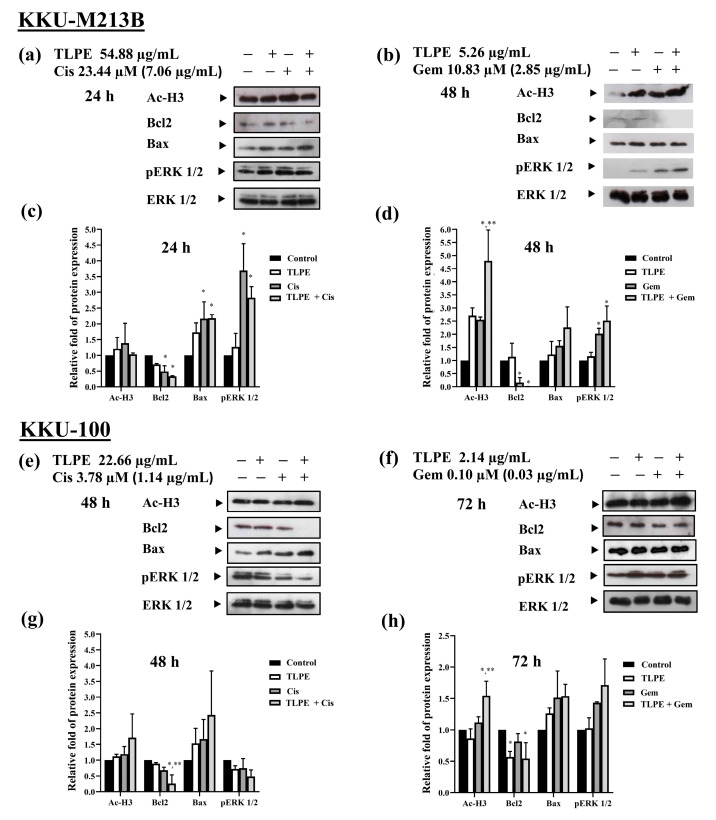
Effect of the combination treatment with TLPE and Cis or Gem on ERK signaling and apoptosis-related proteins. KKU-M213B (**a**,**b**) and KKU-100 (**e**,**f**) cells were treated with the solvent control (0.5% EtOH + 0.5% DMSO, *v*/*v*), TLPE, and Cis or Gem for single and combined agent treatments under the synergistic conditions. In Western blotting, the total ERK 1/2 was employed as a loading control. Densitometric analysis was utilized to calculate the relative protein band intensity. The relative folds of protein expression are displayed as bars for KKU-M213B (**c**,**d**) and KKU-100 (**g**,**h**) cells. The percentages of cells (means ± S.D.) are calculated from two independent experiments performed in duplicate. “*” denotes a statistically significant difference (*p* < 0.05) compared to the solvent control treatment. “**” denotes a statistically significant difference (*p* < 0.05) compared to the single-agent treatments.

**Figure 8:**
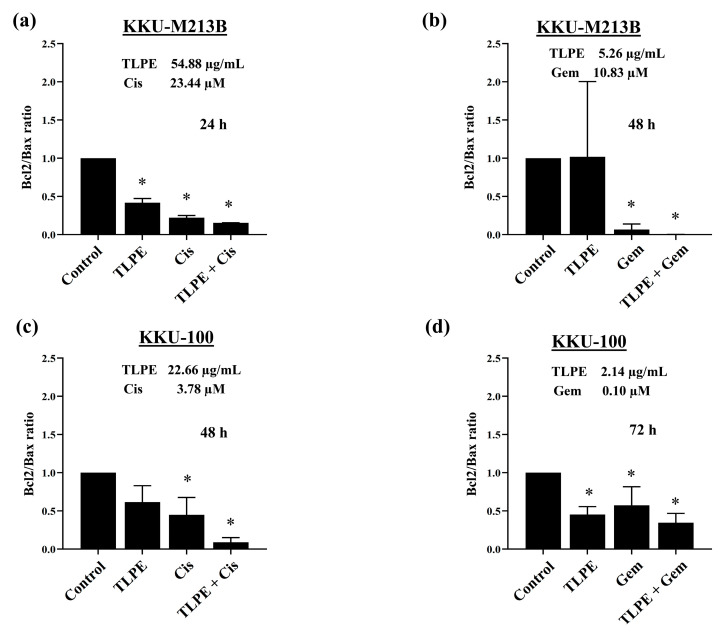
Effect of the combination treatment of TLPE and Cis or Gem on Bcl2/Bax relative expression. KKU-M213B (**a**,**b**) and KKU-100 (**c**,**d**) cells were treated with the solvent control (0.5% EtOH + 0.5% DMSO, *v*/*v*), TLPE, and Cis or Gem for single-agent and combination treatments at the synergistic conditions. The bar graphs show relative folds of Bcl2/Bax expression in KKU-M213B (**a**,**b**) and KKU-100 cells (**c**,**d**). The Bcl2/Bax ratios (means ± S.D.) are calculated from two independent experiments. The symbol “*” indicates a significant decrease when compared with the solvent control treatment (*p* < 0.05).

To understand the molecular mechanisms underlying the anticancer effect of the combination treatment, the pro-apoptosis (Bax), anti-apoptosis (Bcl2), and ERK signaling (pERK1/2) proteins were evaluated using Western blot analysis. In KKU-M213B cells, the combination treatment of TLPE and Cis significantly increased the expression of Bax when compared with the solvent control (Figure 7a,c). In contrast, the levels of Bcl2 were significantly reduced in the combination treatment of TLPE and Cis (Figure 7a,c). In a combination treatment of TLPE and Gem, the level of Bcl2 was significantly decreased while the level of Bax was not significantly changed (Figure 7b,d). The relative ratios of Bcl2/Bax were significantly decreased in the combination treatments of both TLPE with Cis (Figure 8a) and TLPE with Gem (Figure 8b). In addition, the levels of phosphorylated ERK (pERK) were significantly increased in the combination treatments of both TLPE with Cis (Figure 7a,c) and TLPE with Gem (Figure 7b,d).

In KKU-100 cells, the combination treatment of TLPE and Cis caused a significant increase of Bax and a significant decrease of Bcl2 compared with the solvent control treatment (Figure 7e,g). While the combination treatment of TLPE and Gem caused a significant decrease of Bcl2 and caused no significant change in the level of Bax compared with the solvent control treatment (Figure 7f,h). The relative ratios of Bcl2/Bax were significantly decreased in the combination treatments of both TLPE with Cis (Figure 8c) and TLPE extract with Gem (Figure 8d). The combination treatments of TLPE with Cis (Figure 7e,g) and TLPE with Gem (Figure 7f,h) did not cause a significant change in the levels of pERK.

TLPE has been shown to cause hyperacetylation of histone proteins, as demonstrated by upregulation of the Ac-H3 protein in both CCA cell lines at a concentration of 125 µg/mL [28]. In this study, the concentrations of TLPE used in the treatments were less than 125 µg/mL, in which hyperacetylation of histone H3 was not observed in the single agent treatments (Figure 7). However, the combination of TLPE and Gem caused a significant increase in the level of Ac-H3 protein in both KKU-M213B (Figure 7b,d) and KKU-100 (Figure 7f,h) cells.

### 3.8. Antitumor Effect of Cis, Gem, and TLPE on CCA Xenograft Mice

**Figure 9:**
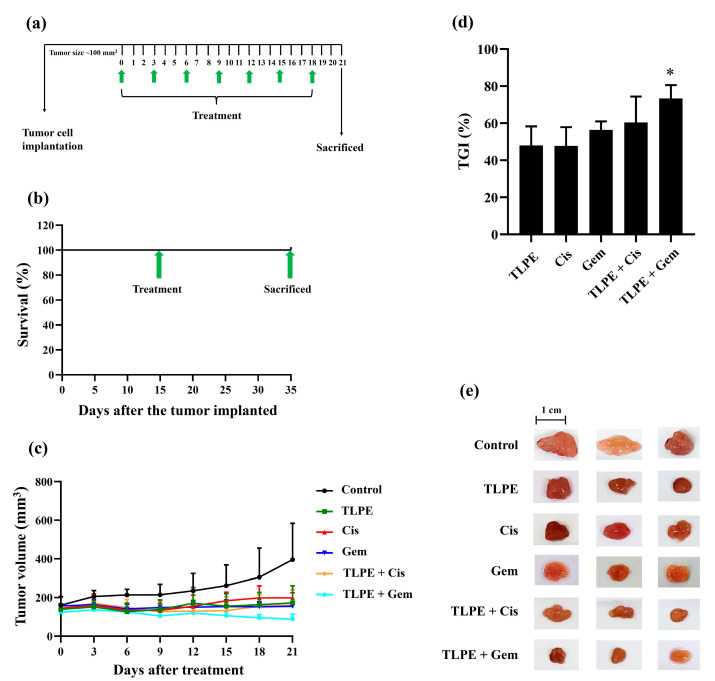
Effects of TLPE, Cis, and Gem alone or in combination on the KKU-100 xenograft mice. Experimental design of the administration of TLPE, Cis, and Gem alone or in combination is shown (**a**). The survival rate of all xenograft mice after treatment is shown (**b**). Tumor volumes of KKU-100 xenograft mice after treatments with TLPE (100 mg/kg), Cis (3 mg/kg), and Gem (100 mg/kg) alone or in combination are shown (**c**). The percentages of tumor growth inhibition after treatments with TLPE, Cis, and Gem alone or in combination are shown (**d**). Representative photographs of tumors are presented (**e**). “*” indicates a significant difference between the single and combined agent treatments (*p* < 0.05).

After the tumor volume reached 100 mm^3^, mice implanted with KKU-100 cells were intraperitoneal (i.p.) injected every three days with DMSO + 1X PBS (1:9) (a vehicle control), Cis, Gem, and TLPE alone or in combination for 21 days (Figure 9a). All mice survived after treatments (Figure 9b). Mice from each group were sacrificed before the tumors were excised and photographed (Figure 9e). The groups of mice treated with single agents of 3 mg/kg Cis, 100 mg/kg Gem, and 100 mg/kg TLPE showed a decrease in tumor volume after 21 days of treatment when compared with the vehicle control group (Figure 9c). Moreover, the groups treated with TLPE in combination with Cis or Gem showed a greater decrease in tumor volume when compared to the groups treated with a single agent (Figure 9c). In addition, tumor growth inhibitions in mice treated with Cis, Gem, and TLPE were 47.78 ± 10.12%, 55.20 ± 4.55%, and 47.93 ± 10.33% compared with the vehicle control group, respectively (Figure 9d). The inhibition of tumor growth in mice treated with the combination of Cis and TLPE was 60.40 ± 13.95%, whereas inhibition of tumor growth in mice treated with a combination of Gem and TLPE was 73.28 ± 7.28% compared with the vehicle control group (Figure 9d).

### 3.9. In Vivo Toxicological Evaluation

Toxicities of the drugs on xenograft mice during treatments were assessed by monitoring body weight changes, organ weight, and histopathology of organs (liver, kidneys, and spleen). The initial and final body weights are shown in Table 3. The body weights of mice in vehicle control, Gem, TLPE, and a combination of Gem and TLPE groups were increased by 4.83%, 3.85%, 2.92%, and 0.83%, respectively. In contrast, the body weights of mice in the groups treated with Cis alone and in combination with TLPE were decreased by 19.59% and 15.21%, respectively. Furthermore, the organ index was calculated as a ratio between organ weight and body weight. Liver weight was significantly reduced in mice treated with Gem alone. Similarly, the kidney weight of the mice treated with Cis alone was significantly reduced compared to that of the vehicle control group. However, the combination treatments of TLPE with Cis and TLPE with Gem showed no significant effect on kidney weight. Treatment with Cis alone caused a significant decrease in the spleen weight, but treatments with TLPE alone and in combination with Cis had no significant effect on the spleen weight. In contrast, treatment with Gem alone caused a significant increase in the spleen weight when compared to the vehicle control group. The spleen weights were significantly increased in mice treated with a combination of Gem and TLPE compared to the vehicle control and Gem alone treatments.

**Figure 10:**
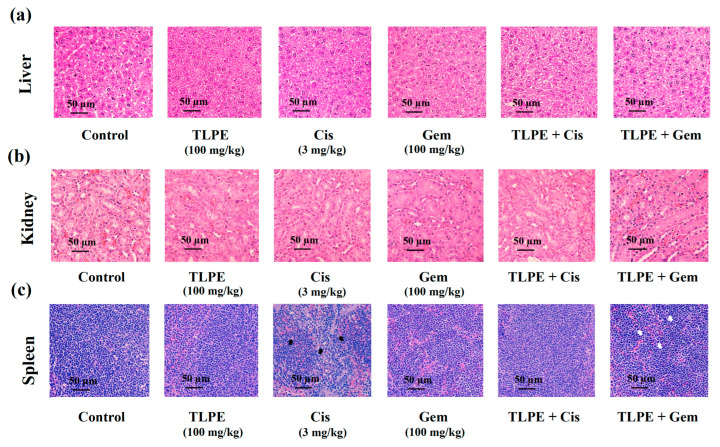
Photomicrographs of hematoxylin and eosin-stained mouse organ sections. The tissues, liver (**a**), kidney (**b**), and spleen (**c**), were stained by hematoxylin and eosin. Nuclear debris and vacuoles are observed in the spleen of mice treated with cisplatin alone (black arrows). The larger spleen cells are observed in the spleen of mice treated with a combination of TLPE and Gem (white arrows). Scale bar = 50 μm.

In addition, the histological sections of liver and kidney tissues showed no significant differences between alone and in combination treatments compared with a vehicle control treatment (Figure 10a,b). Nevertheless, the spleens of mice treated with Cis alone showed some apoptotic and necrotic cells, periarterial lymphatic sheath of the spleen, and vacuoles in the splenic corpuscle, which were found minimally in the combination treatments (Figure 10c). Moreover, treatments with Gem alone and in combination with TLPE caused some spleen cells to be larger in size compared to the control group (Figure 10c).

## 4. Discussion

In this study, TLPE phenolic profiles, total phenolic content, anticancer activity against CCA cell lines, and toxicity against non-cancer cells and RBCs were investigated in vitro. Furthermore, antitumor and toxicity in mouse organs were demonstrated in vivo. Ethanolic extract of the *T. triandra* leaf power showed potent antiproliferative activity against KKU-M213B (IC_50_ = 25.59 ± 2.21 µg/mL) and KKU-100 (IC_50_ = 37.53 ± 5.26 µg/mL) cells at 72 h exposure. The non-cancer H69 cells seemed to be sensitive to all anticancer drugs used in this study at longer exposure times (48 and 72 h) (Figure 2). This could be because H69 cells lacking telomerase activity have been grown in many sub-passages, predisposing to senescence and consequently being more sensitive to the treatments [38]. However, TLPE and the drugs used in this study showed no hemolytic toxicity in RBCs (Figure 3). Similar to our results, a previous study demonstrated that *T. triandra* extracts prepared with different solvents exhibited antiproliferative activity against human small-cell lung cancer (NCIH187) and human oral epidermoid carcinoma (KB) cells [25]. The methanolic extract (IC_50_ = 32.15 ± 10.94 µg/mL and 11.93 ± 4.52 µg/mL) and the water extract (IC_50_ = 2.06 ± 0.84 µg/mL and 12.27 ± 2.98 µg/mL) showed potent antiproliferative activity against the KB and NCIH187 cells, respectively [25]. However, the petroleum ether and the dichloromethane extracts appeared to be inactive in all tested human cancer cells (IC_50_ > 50 µg/mL) [25]. In addition, the combination treatments of TLPE and Cis or Gem showed greater anticancer activity against CCA cell lines than the single-agent treatments (Figure 4). The critical parameters to consider as quantitative indicators of the cancer treatment modality are CI and DRI values. These parameters are very important for figuring out whether the effect of two drugs together on cancer cells is additive (CI = 0.90–1.10), synergistic (CI < 0.90), or antagonistic (CI > 1.10) [37]. In KKU-M213B cells, the combinations of TLPE and Cis indicated a synergism at 24 h exposure (CI = 0.68 ± 0.07), while the combinations of TLPE and Gem indicated a slight synergism at 48 h exposure (CI = 0.84 ± 0.23) (Table 2). These synergistic effects resulted in a dose reduction for Cis and Gem of 3.11- and 1.86-fold in KKU-M213B cells, respectively. In KKU-100 cells, the combination treatments of TLPE and Cis exhibited synergistic drug interactions at 24 h (CI = 0.86 ± 0.04) and 48 h (CI = 0.56 ± 0.08) exposures, while the combination treatments of TLPE and Gem showed synergism at 72 h exposure (CI = 0.63 ± 0.09) (Table 2). These synergistic effects resulted in a dose reduction for Cis (2.11- and 4.05-fold at 24 and 28 h exposures, respectively) and Gem (1.80-fold) in KKU-100 cells. Accordingly, the TLPE exhibited a promising drug combination to enhance CCA inhibition.

*T. triandra* crude extract may contain several bioactive compounds that promote cancer cell inhibition. The phytochemical composition of TLPE contains *p*-coumaric, ferulic, *p*-hydroxybenzoic, sinapinic, syringic, and vanillic acids (Figure 1), which are the same phenolic acids identified in the previous lot of *T. triandra* leaf powder [29]. Consistently, the phenolic acids in *T. triandra* were previously identified as *p*-coumaric, ferulic, gallic, protocatechuic, *p*-hydroxybenzoic, sinapinic, syringic, and vanillic acids [39]. Our previous studies demonstrated that *p*-coumaric, ferulic, and sinapinic acids inhibited the growth of human breast (MCF-7), cervical (HeLa), and colon (HT29 and HCT116) cancer cell lines in vitro [35,40]. The total phenolic content of TLPE determined in this study was 42.51 ± 6.84 mg gallic acid equivalent/g of dry extract compared with the total phenolic content of *T. triandra* extract as 26.70 mg gallic acid equivalent/g of dry extract in a previous study [41]. In addition, oxoanolobine was isolated and identified from *T. triandra* leaves; however, its antiproliferative activity was less effective than some crude extracts (IC_50_ > 50 µg/mL) against oral cavity cancer (KB) and breast cancer (MCF-7), IC_50_ = 27.6 ± 4.30 µg/mL against lung cancer (NCIH187)) [25].

The combination of TLPE and Cis or Gem inhibited the growth of CCA cell lines through cell cycle arrest and induction of apoptosis. Treatment with Cis caused an increase in the sub-G1 population and induced cell cycle arrest at the S phase (Figure 5). This may be one of the direct responses of cells to the DNA damage caused by cisplatin. Moreover, the sub-G1 population was increased in the combination treatment with Cis or Gem and TLPE. Therefore, apoptosis induction was confirmed and demonstrated by dot plots in apoptosis analysis (Figure 6). Consistently, Cis induced cell cycle arrest at the S phase in the previous study [18]. Moreover, Cis was also used in a combination treatment with the plant products, resulting in an increase in the sub-G1 population [18]. The combination treatment of TLPE and Cis or Gem caused an induction of apoptosis via down-regulation of Bcl2 and up-regulation of Bax in both CCA cells (Figure 7). Likewise, apoptosis induction in CCA cells was associated with a decrease in the ratio of Bcl2/Bax in both alone and combination treatments (Figure 8). Similarly, the Bax level was up-regulated while the Bcl2 level was down-regulated in SW1990 and BxPC3 cells treated with Gem and *Clinacanthus nutans* Lindau extracts [42]. The presence of phenolic compounds in various plant extracts was associated with the inhibition of cancer cell growth through increased Bax and decreased Bcl2 expression [43]. The increase of Bax (pro-apoptosis) is commonly associated with intracellular stress from DNA damage or oxidative stress and is required for the release of cytochrome c. Conversely, the decrease of Bcl2 expression involves the release of cytochrome c into the cytosol [44]. Our results indicated that combination treatment with TLPE and Cis or Gem induced apoptosis via intrinsic pathways.

In eukaryotic cells, the ERK/MAP-kinase pathway plays important roles in many signal transduction pathways [45]. In KKU-M213B cells, the combination treatment with TLPE and Cis or Gem caused a significant increase in the level of pERK1/2 (Figure 7). Similar to our results, a combination treatment of Cis and peanut testa extract also caused an increase in the level of pERK1/2 in KKU-M213B cells [18]. This could be explained by apoptosis induction via the generation of reactive oxygen species (ROS) through pERK1/2 up-regulation in a specific cellular context. ROS plays a crucial role in regulating normal physiological function by activating various signaling pathways in a cell. An excess of ROS can cause damage to membranes, proteins, and intracellular organs, which can eventually lead to apoptosis [46]. Furthermore, Ac-H3 was up-regulated in a combination treatment of Cis and TLPE in KKU-100 cells and a combination of Gem and TLPE in both KKU-M213B and KKU-100 cells (Figure 7). It has also been reported that some phenolic compounds with HDAC inhibitory activity induced Ac-H3 expression in breast and cervical cancer cells [40]. The use of HDAC inhibitors in combination treatments with Cis seems to enhance anticancer efficacy and reduce cytotoxicity to normal cells [47,48,49].

A nude mouse CCA xenograft model was used to investigate the possibility of the clinical application of Cis or Gem in combination with TLPE (Figure 9). TLPE enhanced tumor suppression of both Cis and Gem in the nude mice with 100% survival. The in vivo treatment with a combination of Gem and TLPE significantly inhibited tumor growth compared to the single-drug treatment. Based on toxicological evaluations conducted in vivo, toxicity was defined as a toxic death or body weight change (BWC) loss of more than 20% [50]. Our results showed that the mice treated with Gem and TLPE alone or in combination had no changes in their body weights. In contrast, mice treated with Cis alone and a combination of Cis with TLPE had a reduction of 19.59% and 15.21% of their body weight change, respectively (Table 3). According to this result, the treatment of Cis in combination with TLPE enhanced the body weight change of the treated mice compared to those treated with Cis alone. Our finding suggests that Cis alone treatment may suppress the host immune and defense systems, but the combination treatment fosters a decrease in toxicity [51]. The liver weights of mice treated with Gem alone were significantly decreased (Table 3), indicating that treatment with Gem alone caused hepatotoxicity [52]. However, the combination treatment of Gem and TLPE caused no hepatotoxicity (Table 3). Cis has a variety of adverse effects, one of which is that it causes acute kidney injury (AKI) in more than 30% of patients [53]. The toxicity of Cis causes a high mortality rate and has been reported to cause damage to multiple organs [54]. Numerous reports indicate that increased levels of intracellular ROS and inflammatory cytokines are involved in the cytotoxicity associated with AKI [55]. In a previous study, plant-derived bioactive compounds with free radical scavenging activity inhibited and ameliorated ROS-induced oxidative damage in several pathophysiological conditions [56]. In this study, treatment with Cis alone significantly reduced the kidney weight; however, the combination treatment with TLPE restored the kidney weight significantly. Accordingly, TLPE in combination treatment with Cis may protect the kidney from Cis-induced oxidative damage. In addition, treatment with Cis alone significantly decreased the spleen weights of mice, but a combination treatment of Cis and TLPE caused no changes in the spleen weights. The decrease in spleen weight may be explained by the apoptotic effect of oxidative and inflammatory stress on spleen cells [57]. According to histopathological images in Figure 10, the spleens of mice treated with Cis alone show nuclear debris and vacuoles (black arrows). In contrast, treatments with Gem alone and in combination significantly increased the spleen weights. Moreover, the combination of Gem and TLPE stimulates an enlarged spleen, more evident than the single-agent treatments (white arrows). The enlargement of the spleen may be explained by the presence of edema, tissue congestion, less connective tissue, and vacuoles in the tissue [58]. Some drugs promote the enlargement of the spleen via hemolysis and the resulting venous congestion from a liver disorder with portal vein occlusion. However, the spleen can return to its normal size following the cessation of this treatment [59,60,61].

## 5. Conclusions

The findings of this study suggest that TLPE enhances the anticancer activity of Cis and Gem and reduces their toxicity both in vitro and in nude mouse xenograft models.

## Figures and Tables

**Table 1:** Phenolic acid compositions of TLPE.

Sample	Phenolic Acids (mg/g of Dry Extract) ^a^					
	*p*-Hydroxybenzoic Acid	Vanillic Acid	Syringic Acid	*p*-Coumaric Acid	Ferulic Acid	Sinapinic Acid
TLPE	4.07 ± 0.64	2.49 ± 0.58	4.40 ± 1.96	6.65 ± 3.31	5.98 ± 1.34	9.98 ± 2.25

^a^ Results are expressed as means ± SD of two determinations.

**Table 2:** CI and DRI of the combination treatments with TLPE and Cis or Gem against CCA cells.

CCA Cells	Drug Combination				Exposure Time	CI	DRI		
							Cis	Gem	TLPE
	IC_50_ of TLPE (µg/mL)		IC_20_ of Cis (µM)	IC_20_ of Gem (µM)					
	Alone	Combination							
KKU-M213B	204.18 ± 3.47	54.88 ± 10.34	23.44 ± 0.30	-	24 h	0.68 ± 0.07	3.11	-	3.79
	26.06 ± 1.79	16.42 ± 1.05	5.26 ± 0.07	-	48 h	0.98 ± 0.02	4.63	-	1.59
	25.59 ± 2.21	29.37 ± 7.97	3.93 ± 1.27	-	72 h	2.00 ± 0.52	2.72	-	1.15
KKU-100	674.76 ± 6.21	261.60 ± 11.78	23.17 ± 1.43	-	24 h	0.86 ± 0.04	2.11	-	2.58
	91.18 ± 7.51	22.66 ± 5.00	3.78 ± 1.36	-	48 h	0.56 ± 0.08	4.05	-	3.48
	37.53 ± 5.26	17.92 ± 2.73	1.09 ± 0.19	-	72 h	1.04 ± 0.08	2.69	-	2.14
KKU-M213B	26.06 ± 1.79	5.26 ± 4.97	-	10.83 ± 1.97	48 h	0.84 ± 0.23		1.86	8.65
	25.59 ± 2.21	19.61 ± 2.89	-	4.31 ± 0.48	72 h	1.75 ± 0.27		1.70	1.25
KKU-100	91.18 ± 7.51	32.95 ± 0.02	-	0.12 ± 0.02	48 h	1.06 ± 0.06		1.97	2.77
	37.53 ± 5.26	2.14 ± 0.77	-	0.10 ± 0.02	72 h	0.63 ± 0.09		1.87	2.77

CI: combination index; DRI: dose reduction index; CCA: cholangiocarcinoma; Cis: Cisplatin, Gem; Gemcitabine, TLPE: *T. triandra* leaf powder ethanolic extract.

**Table 3:** Body weight, % body weight change (%BWC), and relative organ weight of nude mice in the vehicle control and treated groups.

Group	**Initial Body Weight (g)**	**Final Body Weight (g)**	% BWC	**Organ Index (g/100 g Body Weight)**		
				Liver	Kidney	Spleen
Vehicle control	22.53 ± 0.91	23.60 ± 0.53	4.83	7.66 ± 0.22	0.97 ± 0.05	0.64 ± 0.13
Cis	22.72 ± 1.26	18.31 ± 2.21	−19.59	7.09 ± 0.59	0.84 ± 0.03 ^a^	0.32 ± 0.05 ^a^
Gem	22.26 ± 0.96	23.12 ± 0.94	3.85	6.94 ± 0.22 ^a^	0.95 ± 0.04	1.74 ± 0.32 ^a^
TLPE	22.07 ± 0.60	22.71 ± 0.63	2.92	8.39 ± 0.89 ^a^	1.01 ± 0.03	0.70 ± 0.11
TLPE + Cis	23.13 ± 0.76	19.65 ± 2.66	−15.21	7.99 ± 0.25	0.91 ± 0.02 ^a,b,d^	0.42 ± 0.16
TLPE + Gem	22.94 ± 1.27	23.13 ± 1.18	0.83	7.45 ± 0.34	0.94 ± 0.04	2.39 ± 0.25 ^a,c,d^

Results are expressed as mean ± SD from mice (*n* = 5 mice in each group) treated with Cis: Cisplatin 3 mg/kg, Gem: Gemcitabine 100 mg/kg, TLPE: *T. triandra* leaf powder ethanolic extract 100 mg/kg, and combination of TLPE and Cis or Gem at the mentioned concentrations. ^a^ *p* < 0.05 versus vehicle control, ^b^ *p* < 0.05 versus Cisplatin, ^c^ *p* < 0.05 versus Gemcitabine, ^d^ *p* < 0.05 versus TLPE.

## Data Availability

The data presented in this study are available on request from the corresponding author.

## Supplementary Materials

The following supporting information can be downloaded at: https://www.mdpi.com/article/10.3390/medicina59071269/s1, Figure S1: Antiproliferative effects of Gemcitabine against CCA cells at exposure time of 24 h. KKU-M213B (a), KKU-100 (b), and H69 (c) cells were treated with Gem for 24 h at the indicated concentrations.
