# Supplementary material for: Tiliacora triandra Leaf Powder Ethanolic Extract in Combination with Cisplatin or Gemcitabine Synergistically Inhibits the Growth of Cholangiocarcinoma Cells In Vitro and in Nude Mouse Xenograft Models
Source: Medicina (Kaunas). 2023 Jul 7;59(7):1269. doi: 10.3390/medicina59071269 (PMC10386122; doi:10.3390/medicina59071269)
Supplement: Supplementary file 1 [file medicina-59-01269-s001.zip › medicina-2464650-supplementary.pdf]

Article

Tiliacora triandra Leaf Powder Ethanolic Extract in Combination with Cisplatin or Gemcitabine Synergistically Inhibits the Growth of Cholangiocarcinoma Cells In Vitro and in Nude Mouse Xenograft Models

Arunta Samankul [1], Gulsiri Senawong [1], Suppawit Utaiwat [1], Jeerati Prompipak [1], Khanutsanan Woranam [1], Chanokbhorn Phaosiri [2], Banchob Sripa [3] and Thanaset Senawong [1,*]

[1] Department of Biochemistry, Faculty of Science, Khon Kaen University, Khon Kaen 40002, Thailand; s_arunta@kkumail.com (A.S.); gulsiri@kku.ac.th (G.S.); u.suppawit@kkumail.com (S.U.); jeerati.ppk@kkumail.com (J.P.); khanutsanan_w@kkumail.com (K.W.)

[2] Department of Chemistry, Faculty of Science, Khon Kaen University, Khon Kaen 40002, Thailand; chapha@kku.ac.th

[3] WHO Collaborating Centre for Research and Control of Opisthorchiasis (Southeast Asian Liver Fluke Disease), Tropical Disease Research Center, Faculty of Medicine, Khon Kaen University, Khon Kaen 40002, Thailand; banchob@kku.ac.th

* Correspondence: sthanaset@kku.ac.th

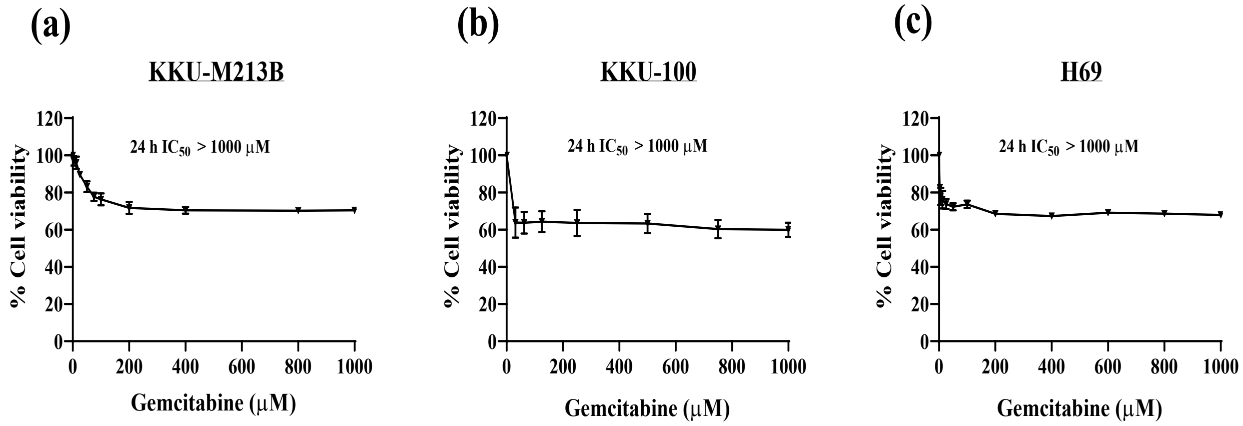

Figure S1. Antiproliferative effects of Gemcitabine against CCA cells at exposure time of 24 h. KKU-M213B (**a**), KKU-100 (**b**), and H69 (**c**) cells were treated with Gem for 24 h at the indicated concentrations.
